# What’s to come after isolation of the pulmonary veins?

**DOI:** 10.1007/s12471-014-0646-5

**Published:** 2015-01-09

**Authors:** Lisette JME van der Does, Natasja MS de Groot

**Affiliations:** Unit Translational Electrophysiology, Department of Cardiology, Erasmus Medical Center, Thorax Center, ‘s Gravendijkwal 230, 3015 CE Rotterdam, The Netherlands

Treatment of atrial fibrillation (AF), the most prevalent tachyarrhythmia worldwide, remains a great challenge. About 15 years ago, when Haissaguerre et al. [1] first described episodes of AF triggered by focal activity originating from the pulmonary veins, the way was paved for ablative therapy of AF by pulmonary vein isolation (PVI). Despite a successful PVI, electric pulmonary vein reconnections still occur frequently requiring additional procedures. To this end new mapping, navigation, imaging and ablation techniques continue to be developed thereby facilitating and increasing success rates of PVI.

In this issue of the *Netherlands Heart Journal*, the results of the endoscopic laser balloon ablation system (EAS) for PVI are presented by Gal et al. [2] in 50 patients with mostly paroxysmal AF (82 %). The EAS provides an elegant manner for optimal circumferential contact and ablation lesions with one device applicable in pulmonary veins of various sizes. Furthermore, an endoscope within the device provides real-time visualisation of the ablation area. This resulted in isolation of 99.5 % of the pulmonary veins during the ablation procedure. However, the long-term results were similar to other techniques and were somewhat lower compared with other studies using the EAS. The authors believe this may be due to inexperience with this novel technology, although other influential factors could be that this study, in contrast to others, also included some patients with persistent AF and had a longer follow-up period. Unfortunately, the study did not report how many pulmonary veins were in fact electrically reconnected with re-ablation procedures. This could provide more information on the long-term success of isolation with the EAS. In addition, it would give insight into the incidence of AF recurrence despite successful isolation of the pulmonary veins. For example, one study [3] using this technique reported AF recurrence in 28 % of patients with persistent pulmonary vein isolation after a median of 12-month follow-up. Moreover, three months after PVI, 14 % of ablated pulmonary veins were reconnected in 38 % of the patients, which entails that a large proportion of patients had at least one pulmonary vein electrically reconnected. We commend the authors for their contribution to optimising PVI outcomes and encourage them in their ongoing efforts to improve the PVI procedure with this refined technique. However, AF recurrences may also have aetiologies outside of our current understanding and perhaps these unknown pathological or physiological processes prevent further improvements in successful long-term outcomes. For this, it is of utmost importance to learn more about the pathophysiology of AF.

The revolutionary findings of Haissaguerre et al. made it possible for a majority of patients with AF to be successfully treated with PVI. Unfortunately, another large proportion of patients continue to have AF, and PVI seems to be insufficient. For this reason different ablation strategies were implemented, such as boxlines, roof, floor and isthmus lines, complex fractionated atrial electrogram ablation and ablation of ganglionated plexi. Nonetheless, AF still recurs and the pathophysiological background to support these additional strategies is controversial.

To take new steps toward successful treatments for AF, we must further understand the underlying mechanism of AF, just as Haissaguerre et al. did almost two decades ago. In previous mapping studies, we demonstrated that longitudinal dissociation in conduction and focal fibrillation waves are the key elements in persistence of AF [4, 5]. Figure 1 shows an example of an epicardial high-resolution wavemap (244 electrode, inter-electrode distance 2.5 mm) of the right atrial free wall obtained from a patient with longstanding persistent AF demonstrating a complex pattern of activation. The wavemap shows individual fibrillation waves represented by colours according to their sequence of appearance. A previously described mapping algorithm was used to classify them intoperipheral waves (entering the mapping area from outside the electrode array), breakthrough waves (appearing at the epicardial surface inside the mapping area) or discontinuous conduction waves (fibrillation waves starting with a delay of 13–40 ms from boundaries of other waves). During this AF episode of *only* 140 ms, the mapping area (diameter: 4 cm) is activated by as many as 15 fibrillation waves, including 5 peripheral waves, 4 epicardial breakthrough waves and 6 discontinuous waves. Hence, when patterns of activation during AF become too complex, the end stage of AF might have been reached and it is likely that PVI in these patients will be unsuccessful. We believe that by understanding the pathophysiology of AF we can come to new successful treatment strategies, which can be used when PVI just is not enough. Presumably, this will entail a tailor-made treatment determined by different mechanisms underlying AF.

**Fig. 1:**
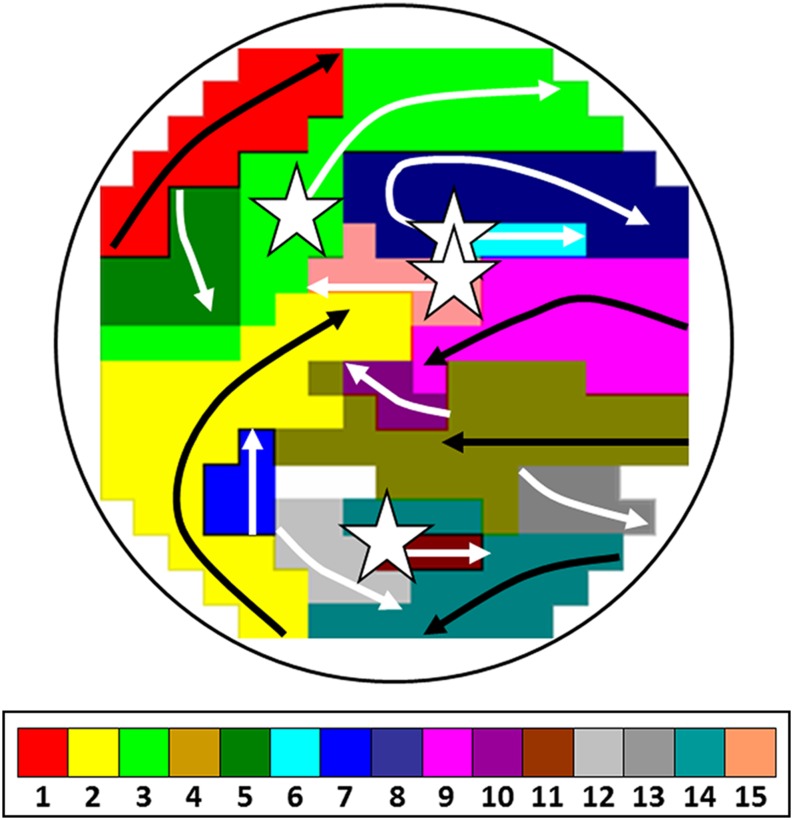
Wavemap constructed during longstanding persistent AF. Sites of epicardial breakthroughs are indicated by *white asterisks*; *white arrows* indicate direction(s) of expansion of epicardial breakthrough or discontinuous fibrillation waves; *black arrows* indicate waves coming from outside the mapping area

## Funding

None.

## Conflict of interests

None declared.
